# 3D printed microfluidic device for online detection of neurochemical changes with high temporal resolution in human brain microdialysate†

**DOI:** 10.1039/c9lc00044e

**Published:** 2019-05-16

**Authors:** Isabelle C. Samper, Sally A. N. Gowers, Michelle L. Rogers, De-Shaine R. K. Murray, Sharon L. Jewell, Clemens Pahl, Anthony J. Strong, Martyn G. Boutelle

**Affiliations:** a Department of Bioengineering, Imperial College, London, UK.; b Department of Basic and Clinical Neuroscience, King’s College, London, UK

## Abstract

This paper presents the design, optimisation and fabrication of a mechanically robust 3D printed microfluidic device for the high time resolution online analysis of biomarkers in a microdialysate stream at micro-litre per minute flow rates. The device consists of a microfluidic channel with secure low volume connections that easily integrates electrochemical biosensors for biomarkers such as glutamate, glucose and lactate. The optimisation process of the microfluidic channel fabrication, including for different types of 3D printer, is explained and the resulting improvement in sensor response time is quantified. The time resolution of the device is characterised by recording short lactate concentration pulses. The device is employed to record simultaneous glutamate, glucose and lactate concentration changes simulating the physiological response to spreading depolarisation events in cerebrospinal fluid dialysate. As a proof-of-concept study, the device is then used in the intensive care unit for online monitoring of a brain injury patient, demonstrating its capabilities for clinical monitoring.

## Introduction

Chemical changes in the body can provide essential information on tissue physiology and pathology and can guide patient treatment protocols.^1,2^ In a clinical setting, these measurements rely on discrete blood samples, which are time-consuming and offer poor temporal resolution. As a result, it is not possible to resolve local changes in analytes such as neurotransmitters or metabolic biomarkers in the immediate aftermath of tissue damage. Current research is focussed on developing tools to accurately measure dynamic changes in these analytes with high temporal resolution in order to aid clinical decision-making and to allow medical intervention to be administered earlier.

Microdialysis is a sampling technique that can be used to locally monitor the metabolic state of tissue. This technique allows collection of molecules from a target tissue, creating a dialysate stream that is analysed *ex vivo* for compounds of interest. Analysis is traditionally carried out offline, in discrete samples, which results in poor temporal resolution. Another consequence of offline analysis is that concentrations are averaged within a sample; for dynamic changes that are fast compared to the sampling period this results in attenuation of the signal. Online microdialysis,^3^ where the dialysate is analysed either continuously or near-continuously using an automated flow-injection sampling method, has been developed to overcome these issues. Combined with online analysis systems, online microdialysis provides high time resolution suitable for resolving dynamic changes in metabolite and neurotransmitter levels.^4–7^ Online microdialysis has been coupled with various high-frequency flow injection analytical techniques including electrochemical biosensors,^8–13^ capillary and microchip electrophoresis^14–16^ and liquid chromatography mass spectrometry.^17–19^ A recent development from flow-injection analysis is to carry out measurements in the dialysis stream continuously, improving the time resolution further still.^20–24^ Due to the availability of commercial FDA-approved probes, online microdialysis has been used in various clinical situations including monitoring of traumatic brain injury,^8,9,24^ bowel ischemia^13^ and free flap surgery.^12^

Microfluidic devices are particularly well suited for online analysis of dialysate streams as they provide the low internal volumes required for high time resolution at the low flow rates typically found with microdialysis (0.3–2 μl min^−1^). Traditionally, microfluidic devices have been fabricated with materials such as PDMS, thermoplastics or glass using soft lithography, micro-thermoforming, micro-milling or injection moulding techniques.^25–27^ Among these methods, PDMS-based soft lithography is still the most commonly used in the field of microfluidics due to its capacity to build very small channels, its low cost and its ease of fabrication. Although PDMS-based microfluidic devices have been used extensively in proof-of-concept studies for a wide range of applications,^28–31^ there are a range of challenges associated with them. Firstly, due to its elasticity, PDMS lacks mechanical robustness and micro-channels made of PDMS can deform under pressure, causing irregular flow, leaks to occur or air bubbles to become trapped, all perturbing the sample stream. This lack of robustness also makes it difficult to build secure and precise connections between PDMS-based microfluidic devices and other components. Therefore, expertise is required to handle and operate PDMS microfluidic devices making them impractical for non-experts, such as clinicians. Secondly, considerable expertise is required to reproducibly manufacture PDMS microfluidic devices in large quantities.

Microfluidic devices are increasingly being produced using additive manufacturing (3D printing) techniques as these offer many advantages over PDMS-based soft lithography.^32,33^ Firstly, 3D printing offers a wide range of available materials, as well as the possibility to integrate multiple materials with different mechanical properties into the same device in order to create a durable assembly that can easily connect to external components. Secondly, 3D printing is a one-step-production process that allows rapid prototyping and, more importantly, reproducibility. Therefore, 3D printing techniques offer a convenient route to scalability, which is essential in order to move from proof-of-concept lab-based systems to more easily manufacturable systems for early clinical trials.

Online microdialysis combined with a 3D printed microfluidic device is particularly applicable for monitoring traumatic brain injury patients in the intensive care unit (ICU), where it is essential to have a sturdy system providing high temporal resolution. The injured human brain spontaneously undergoes spreading depolarisation (SD) events, which are associated with rapid neurochemical changes. An SD consists of a mass depolarisation of all neuronal cells, propagating like a wave over the cortex.^34^ SDs have been associated with the development of secondary damage and have been shown to independently predict poor patient outcome.^35–37^ The electrophysiological and chemical signatures of SDs have been recorded both in animal models^22,38–41^ and clinically.^24,42,43^ The occurrence of an SD wave is reflected in the local tissue by a transient increase in the extracellular levels of potassium and glutamate, coupled with a simultaneous decrease in glucose and increase in lactate, indicating a high energy demand.^43^ When we have used online microdialysis clinically,^22,24^ the metabolic response to an SD appears to take place on a slower timescale and exhibits a lower amplitude than when recorded with small implanted biosensors.^38^ Predominantly this is because the delicate PDMS-based microfluidic analysis system is protected in a metal box on a large trolley behind the patient’s bed and, to allow patient movement, the system is connected to the patient by one metre of low-volume tubing.^24^ However, due to Taylor dispersion, this connection tubing creates dispersive blurring of sharp changes in metabolite concentration,^44^ which in some cases lowers the amplitude of the transient changes below the limit of detection of the sensors, preventing the detection of an SD event.

Here, we describe an improved lab-on-a-chip analysis system that is mechanically robust enough to be positioned closer to a patient and fast enough to measure the dynamic chemical changes associated with SDs, including those of the rapidly changing neurotransmitter glutamate. It consists of a 3D printed microfluidic device with low internal volume to analyse the dialysate stream. It incorporates a system for precisely positioning biosensors for analytes such as glutamate, glucose and lactate in the centre of the dialysate flow stream allowing a fast response^45^ while enabling them to be easily replaced if necessary. The main challenges in building this lab-on-a-chip platform are therefore to incorporate the smallest microfluidic channel possible with secure, reusable low-volume connections for both the dialysate and the sensors. Optimisation of the size of the 3D printed channel and the resulting improvement in analyser response time are presented, together with characterisation of the fastest pulses the device can resolve. As a proof-of-concept experiment, the device is employed within the current system in the ICU for online monitoring of a brain injury patient, demonstrating its capabilities for clinical monitoring.

## Experimental section

### Reagents

Glucose oxidase from *Aspergillus niger* and lactate oxidase from *Aerococcus viridans* were purchased from Sekisui Diagnostics. l-Glutamate oxidase from *Streptomyces* sp. was purchased from Cosmo Bio Co. Polyurethane (Texin 985) was purchased from Bayer. All other chemicals were obtained from Sigma-Aldrich and were of at least ACS reagent grade.

### Electrode fabrication

Biosensors used in this study were based on combined needle electrodes.^22,46^ Briefly, a 50 μm Teflon insulated platinum wire (A-M Systems, Inc) and a 50 μm polyester insulated silver wire (GoodFellow, Inc) were threaded through a 27G hypodermic needle. A flame was used to expose the metal at each end of the wires and the top ends were connected to contact pins *via* conventional electrical wires using conductive silver epoxy (RS Components Ltd). The internal volume of the needle was filled with epoxy resin (Robnor Resinlab Ltd) and left to cure at room temperature overnight. The sharp end of the needle was then cut and sanded down to expose the microelectrode discs at the tip. The blunt needle was then polished further with alumina slurries (1, 0.3 and 0.05 μm). The platinum disc acted as the working electrode and the silver disc was made into a Ag|AgCl reference electrode by dipping the needle tip in a chloridising solution (BASi) for 5 s and then in a 1 M hydrochloric acid solution for 20 s. The needle shaft was connected to an electrical wire using conductive silver epoxy glue and acted as the counter electrode. The working electrode surface was assessed using cyclic voltammetry.

### Biosensor fabrication

**Fig. 1:**
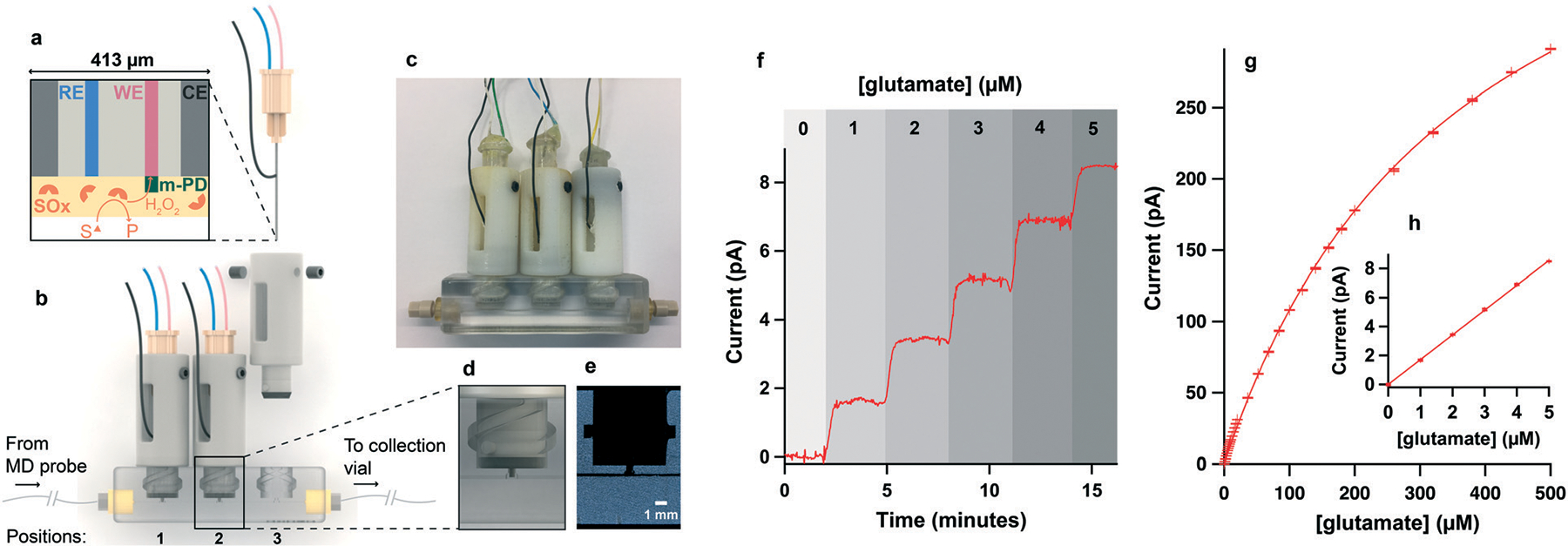
Microfluidic biosensing platform. a. Schematic of combined needle electrode with cross-section of the needle tip showing reference (RE), working (WE) and counter electrodes (CE) to give a complete electrochemical cell. To make this electrode into a biosensor, a poly(*m*-PD) exclusion layer and a hydrogel layer containing substrate oxidase (SO_*x*_) are added. b. Schematic of combined microfluidic platform showing needle biosensors mounted into 3D printed holders and secured into a 3D-printed microfluidic channel through which the dialysate flows. Labsmith CapTite bonded-port connectors for inlet and outlet junctions are represented in yellow. c. Photo of combined biosensing microfluidic platform. d. Zoomed-in image showing biosensor tip positioned in the microfluidic channel. e. Computed tomography (CT) scan image of 3D printed channel. f. Current recorded for a glutamate biosensor mounted in position 1 of the chip for a 6-point glutamate calibration from 0 to 5 μM in 1 μM steps at 2 μl min^−1^. Savitzky–Golay filter over 51 points. g. 31-point calibration curve for typical glutamate biosensor in 2 μl min^−1^ flow. h. Zoom in of low concentration range corresponding to data shown in f. In g. and h. The markers represent the mean and the error bars indicate the standard deviation of 1 minute of the plateau current shown in f. The detection limit of this sensor (corresponding to 3 times the standard deviation of the 0 μM plateau current) is 0.25 μM.

Our fabrication method uses hydrogels to give high enzyme loading.^47^ To fabricate biosensors, two layers were systematically added onto the electrode surface, as shown in Fig. 1a. Firstly, a protective poly(*m*-phenylenediamine) (poly(*m*PD)) film was electropolymerised onto the working electrode surface by placing the needle tip into a 100 mM solution of *m*PD in 10 mM PBS at pH 7.4 and holding the working electrode at 0.7 V for 20 minutes. Cyclic voltammetry was used to assess the coating. A second layer consisting of a hydrogel was then added to immobilise enzymes onto the electrode surface, as described elsewhere.^47^ Full details are given in the ESI.† For the detection of SD events, a third layer of polyurethane was added onto the lactate biosensors in order to extend their dynamic range to higher lactate concentrations. In this study, biosensors do not have this additional layer unless specified.

### Recording unit

In-house high-performance potentiostats connected to a PowerLab (ADInstruments) were used to hold the working electrode at +0.7 V *vs*. Ag|AgCl and to record biosensor output currents. All signals in this study were recorded with a sampling frequency of 200 Hz and a 10 Hz low-pass filter.

### Microdialysis probes

Both clinical and in-house microdialysis probes were used. For clinical studies, a CMA70 brain microdialysis probe (10 mm length membrane, 600 μm diameter, 20 kDa molecular weight cut-off (MWCO), MDialysis, Sweden) was used. For *in vitro* studies, in-house concentric microdialysis probes (10 mm membrane length, 280 μm diameter, 13 kDa MWCO) were constructed using Spectra/Por microdialysis hollow fibres. Full details of the fibres used are given in the ESI.†

### Soft PDMS microfluidic chip fabrication

PDMS chips were fabricated using PDMS silicone elastomer and curing agent (Sylgard 184, Dow Corning) in the ratio 9 : 1. The mixture was poured onto a positive SU8 master (200 μm width, 100 μm height channel, fabricated using soft lithography) and placed in the oven at 65 °C for 1 h, after bubbles were removed using a desiccator. Once cured, holes were pierced for the inlet/outlet tubing and for the sensors. The base of each chip was made by semi-curing the PDMS mixture before assembling the two parts and placing them in the oven at 65 °C overnight to fully cure.

### Hard microfluidic chip 3D printing

In this study, two different printers using distinct technologies were tested to print the hard microfluidic flow-cells: the Ultra 3SP and the Objet 30. The Ultra 3SP uses a scan, spin and selectively photocure technology, which projects images from a digital light source to selectively cure a vat-contained photopolymer voxel by voxel. It provides 100 μm resolution in the *x*- and *y*-directions and 50 to 100 μm resolution in the *z*-direction according to its technical datasheet. However, the true resolution depends on several other parameters such as the printing material used and the geometry of the part. Here, the material loaded in the Ultra 3SP was the “ABS white”, which is known for its toughness and mechanical stability. The second printer tested to print the microfluidic chips was the Objet 30, which uses Polyjet technology. This involves jetting layers of curable liquid photopolymer onto a build tray and instantly curing them with UV. To build intricate features such as a microfluidic channel, it deposits soluble support material (SUP706), which is removed afterwards by bathing the part in a 2% (w/v) sodium hydroxide solution. This printer has an accuracy of 100 μm, although this can vary depending on the geometry of the part, the size, orientation and material used. For our microfluidic chips, the material used with this printer was VeroClear (RGD810), which allows transparency as well as dimensional stability and surface smoothness.

### 3D printed chip post processing

The chips 3D printed with the Ultra 3SP were rinsed with IPA and water. They were then placed in a UV oven for 30 s on each side. This post-curing stage was carried out to make the chips harder and more stable, especially around the sensor inserts, which were subjected to friction. The chips printed with the Objet 30 were first cleaned with a water jet to remove most of the supporting material. The parts were then soaked in a 2% sodium hydroxide solution for 2 hours and finally rinsed with water. This process could be repeated several times if needed. When supporting material still remained in the channel, a 70 μm tungsten wire was used to help extrude it.

### 3D printing of electrode holder

Electrode holders were 3D printed with the Objet 260 Connex 3D printer because it can print materials with different rigidity on the same component. Here, the main part was made from hard VeroWhitePlus (RGD835) and the softer tip was made of TangoBlack (FLX973). Once printed, the holders were washed with a water jet to remove all supporting material. Then, the electrode hole was cleared using a 30G needle. The electrode was positioned inside the holder using a cross-sectional cut-out of the chip and secured in place by two M2.5 side screws (Fig. 1b). This was to ensure that once the holder was locked in the chip, the electrode tip would be placed in the middle of the electrode chamber, which consisted of a wider opening along the microfluidic channel that could fit the 27G electrode tip.

### Microfluidic workstation

Sensor calibration and testing was done using an automated microfluidic workstation^52^ built with LabSmith components. It consisted of a computer-programmed bread-board mounted with two 20 μl pumps, two 1 ml reservoirs and two 3-way valves. Each reservoir contained a different concentration of the analyte(s) of interest. By varying the flow rate of each pump, different analyte concentrations were generated while maintaining a constant total flow rate of 2 μl min^−1^.

### Microfluidic tubing

The microfluidic workstation was assembled using polyetheretherketone (PEEK) 150 μm i.d., 360 μm o.d. tubing (Kinesis). The extension tubing used for the microdialysis experiment was fluorinated ethylene propylene (FEP) 120 μm i.d., 1/32″ o.d. tubing (Kinesis). The same extension tubing (one metre long) was used for the clinical experiment.

### Patient recruitment

Patients requiring emergency craniotomy for the treatment of vascular and traumatic brain injuries were identified and research consent was obtained from legally authorised representatives. Study data was anonymised and securely stored. All human research procedures were approved by the appointed UK Research Ethics Committee and were conducted in accordance with the Declaration of Helsinki.

### *In vivo* microdialysis probe insertion

Upon completion of surgery, a clinical microdialysis probe was inserted into the cortex. The aim was to place the probe in the *peri*-lesion tissue around the injury core. The probe was externalised through the scalp and secured using double stay sutures.

### Patient monitoring

In addition to the microdialysis data, electrocorticography (ECoG) data was also collected as previously described.^24^ A continuous 21-channel EEG recording was also acquired. Arterial blood pressure was continuously recorded and blood gases, glucose and electrolytes were documented periodically.

### Postoperative care in the ICU

Patients were sedated and treated according to King’s College Hospital protocols.

## Results and discussion

### 3D printed microfluidic platform design

The microfluidic platform design was based on a device developed by Gowers, Curto *et al*.,^23^ which, though lacking the necessary temporal resolution for this application, provides a secure way of inserting sensors into the microfluidic channel. It consists of two distinct parts: the biosensor-enclosing holders and the microfluidic chip (Fig. 1b and c). The holders are locked in the microfluidic chip using two side pegs that slide into matching guiding slots (Fig. 1d). The holders have a soft plastic annulus (made of TangoBlack material) at the bottom that compresses when the holder is locked into the microfluidic flow-cell, providing a leak-free connection between the sensor and the dialysate stream. In our experience, the connection of the microdialysis probe outlet to the channel is critical for high temporal resolution. Initially, attempts were made to 3D print or tap the thread of a 1/32″ one-piece fitting (2–56 UNC LabSmith) directly into the microfluidic chip, however, this solution was only found to be effective for the first use. Leaking was observed after 1–2 uses as the thread started to erode. To overcome this issue, CapTite bonded-port connectors from LabSmith were inserted into a matching 3D printed aperture at the inlet and outlet of the channel (Fig. 1b), providing a secure low volume connection and allowing collection of the dialysate at the chip outlet for further analysis if required.

Typical sensor current responses to computer-generated concentrations of substrate at 2 μl min^−1^ are shown in Fig. 1f–h for a glutamate biosensor.

### Microfluidic channel optimisation

Two 3D printers (Ultra 3SP and Objet 30) were tested to optimise fabrication of the microfluidic channel. For each printer, the microfluidic flow-cell was printed with different channel dimensions and in different orientations in order to obtain the best spatial resolution. Indeed, one of the biggest challenges in 3D printing microstructures is that the dimensions of the printed parts differ from that specified in the design file. For the Objet 30, an inkjet printer, this deviation from the specified dimensions was mostly caused by the asymmetry of the part being printed; creating an uneven distribution of weight around the microchannel that resulted in distortions. For the Ultra 3SP, a stereolithography (SLA) printer, the vat of liquid polymer precursor around the part helped support the weight; in this case dimension errors mainly occurred due to shrinkage of the ABS white during cooling. To address these issues, a CT scan (Skyscan 1272, Bruker) was used to assess and measure the printed channel. The design files were then amended depending on actual dimensions achieved with each printer/orientation and reprinted iteratively to optimise the channel spatial resolution. For cost and time effectiveness, this optimisation process was first done on shorter microfluidic chips containing one biosensor insert only. The smallest functional microfluidic channels obtained from both the Objet 30 (chip 1) and the Ultra 3SP (chip 2) are featured in Table 1 together with their printing orientation, their actual dimensions and the dimensions specified in the design file. For comparison, Table 1 also presents these parameters for the device previously developed by Gowers, Curto *et al*.^23^ (chip 3).

Ideally, the microfluidic channel should be circular to provide minimum dispersion. Both rectangular and circular channels were designed and compared in this study. Channels that were circular in the design file were found to collapse more frequently than rectangular ones. Furthermore, prints for rectangular channels appeared in practice with rounded corners. Therefore, the design dimensions presented in Table 1 are for rectangular channels only.

For the Ultra 3SP, the optimum printing orientation was found to be with the channel built along the vertical *z*-axis. This was to be expected, as accuracy is always greater in the horizontal *xy*-plane. However, for the Objet 30, printing the chip with the channel vertical resulted in distortions along the channel and around the sensor inserts, which appeared flattened. These effects were accentuated when printing the longer chips required for more than one sensor, and were presumably due to insufficient or uneven support around the vertical axis. This issue was resolved by printing the chip with the channel horizontal and accounting for the lower accuracy of the *z*-direction in the design file dimensions. Typically, the channel height defined in the design file had to be 20% larger than the one aimed at in the final print to compensate for this shrinkage. Printing the channel in the *xy*-plane helped to minimise feature deformations.

**Fig. 2:**
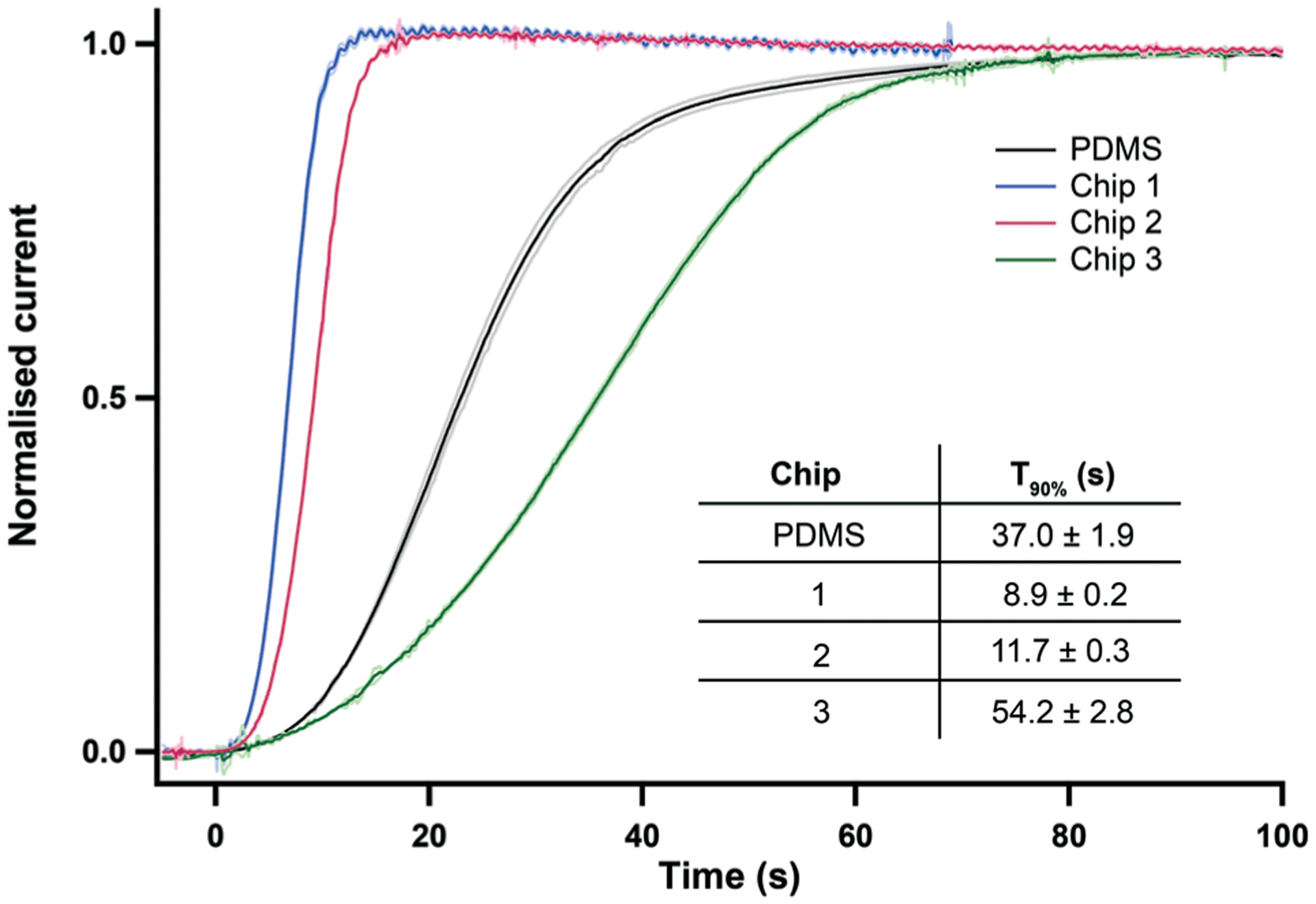
Normalised current response for a 0 to 2 mM lactate step change at 2 μl min^−1^. Data is from the three 3D printed microfluidic chips (position 1) referred to in Table 1 and from our gold-standard PDMS chip. The graph shows the average response of 5 repeats for each chip. The mean ± standard deviation is shown in lighter colours for each trace. Inset table shows the 90% response time *T*_90_ (mean ± standard deviation, *n* = 5).

These chips were then tested to study the effect of the channel size on the sensor response time to a change in concentration. As chip dimensions vary between different materials and different printing orientations, the design of the sensor holder was adapted for each flow-cell in order to ensure a leak free connection and to run a fair comparison test. Fig. 2 shows the current responses of a lactate biosensor recorded at 2 μl min^−1^ in the three different 3D-printed chips featured in Table 1, as well as in the standard PDMS chip currently used for clinical monitoring^24^ shown in Fig. S1.† The measurement set-up (including the microfluidic tubing used) was kept constant across the four chips tested.

The sensor responses recorded in the optimised 3D printed chips 1 and 2 were extremely reproducible as shown by the standard deviation traces in Fig. 2. The reproducibility of the signal was greater for these chips than for the PDMS chip, presumably due to the latter being soft and compliant. Both chips 1 and 2 also provide a channel that is far smaller than that of chip 3. This improvement is reflected in the *T*_90_ time response of the sensing platform being more than 3 times smaller for chips 1 and 2 than for chip 3, as seen in the inset table of Fig. 2. The sensor responses recorded in chips 1 and 2 also show a huge improvement in the speed of response compared to our standard PDMS channel, with the *T*_90_ response time reduced by 68% for chip 2 and 76% for chip 1. The slow response of the PDMS chip reflects both the compliance of PDMS chip (leading to expansion of channel dimensions) and the great practical difficulty of making near zero dead volume connections between tubing and channels in the PDMS.

Of the two printers tested, the smallest channel was achieved with the Objet 30 giving the fastest response time to the 2 mM lactate step with a *T*_90_ of 8.9 ± 0.2 s at 2 μl min^−1^ (chip 1). However, these tests were run in shorter flow cells made for one sensor only. Longer versions of this channel were found to be extremely hard to clear of the supporting material filling. In general, the post-printing cleaning stage required for the Objet 30 was not only very long and tedious but critically also lacked accuracy and reproducibility, resulting in variability across channel sizes and shapes (data not shown). This was due to the fact that the aspect ratio of this channel was too great for the supporting material to be dissolved by soaking the part in a sodium hydroxide solution. Thus, tungsten wire was used to push the resin out of the channel, which resulted in irregularities along the channel walls. Tungsten was chosen for its hardness but due to the very small diameter needed to fit the channel, the wire was still bendable and often generated a non-straight channel. An example of this is shown in Fig. S2.† Therefore, even though the Objet 30 produced the chip with the smallest microfluidic channel, the printer chosen to print our flow cell was the Ultra 3SP because it required minimal post-processing; no removal of supporting material from the channel was required, making the process far quicker and, importantly, more reproducible. Furthermore, due to the liquid vat supporting the part during printing, no distortion was observed along the channel, even for a chip that contained up to 4 sensor inserts. The channel printed was very neat, as seen on the CT scan image of Fig. 1e, and exhibited a smooth surface with rounded corners. A cross section of this quasi-circular channel is shown in Fig. S3.† The flow cell chosen to be used for the rest of this study is therefore chip 2.

### Characterisation of dynamic events using 3D printed chip

**Fig. 3:**
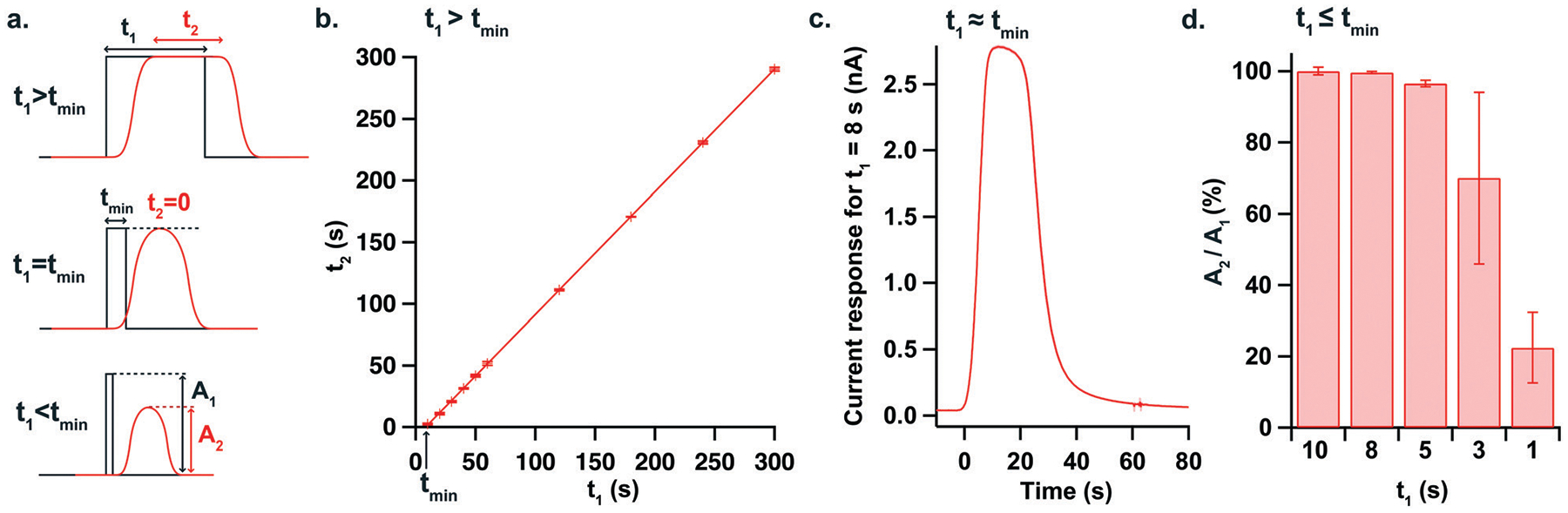
Characterisation of a dynamic change in microfluidic chip 2. a. Diagram showing the effect of Taylor dispersion down the microfluidic channel. The black line represents a concentration pulse change lasting *t*_1_ generated upstream while the sensor response observed downstream (hence with a slight delay) is shown in red. As *t*_1_ decreases, the plateau duration *t*_2_ observed at the sensor decreases until it reaches 0 when *t*_1_ equals *t*_min_, the minimum pulse duration where the full magnitude of the change can be resolved. When *t*_1_ is less than *t*_min_, the amplitude of the change measured at the sensor (*A*_2_) becomes smaller than the amplitude of the pulse generated upstream (*A*_1_). b. Lactate concentration pulses from 0 to 2 mM of different durations (*t*_1_) were generated at a flow rate of 2 μl min^−1^ using the microfluidic workstation and the current was recorded for a lactate biosensor placed in the microfluidic chip (position 1). The plot shows the plateau duration *t*_2_ recorded at the sensor for each pulse duration *t*_1_ generated, provided that the full change magnitude was reached. Each point represents the mean ± standard deviation for 3 repeats. The equation of the fitted line (*R*^2^ = 0.99998) is *t*_2_ = 0.99 × *t*_1_ − 8.35, giving *t*_min_ = 8.38 ± 0.25 s. c. Current response recorded for a lactate biosensor for the shortest pulse that can be fully resolved with this chip (8 s). The red trace shows the mean ± standard deviation for 3 repeats. d. Amplitude of current responses for *t*_1_ < *t*_min_. Amplitudes are expressed as a percentage of the full change magnitude, taken for *t*_1_ = 10 s. Each bar represents the mean for 3 repeats. The graph shows that pulses as short as 5 s are resolved at more than 95%.

To further characterise the temporal resolution of the sensing platform, 2 mM lactate pulses of different durations were generated with our high-precision microfluidic workstation and current responses were recorded for a lactate sensor placed in position 1 of the analysis chip. Due to Taylor dispersion acting along the microfluidic channel, concentration changes recorded downstream at the sensors were broadened compared to the upstream pulses, as illustrated in Fig. 3a. Thus, there is a minimum pulse duration (*t*_min_) below which the current response will not reach its full amplitude. To find this threshold, the plateau durations of the current responses were plotted against the durations of the corresponding generated pulses, as shown in Fig. 3b. The minimum pulse duration fully resolved by the sensing platform was then deduced from the equation of the fitted line and was found to be 8.38 ± 0.25 s. The current response to an 8-second pulse is shown in Fig. 3c and represents the most dynamic change that the platform can fully resolve. Fig. 3d shows the amplitudes of responses to shorter pulses, demonstrating that pulses as short as 5 s can still be resolved at more than 95% of full magnitude.

### *In vitro* SD simulation

**Fig. 4:**
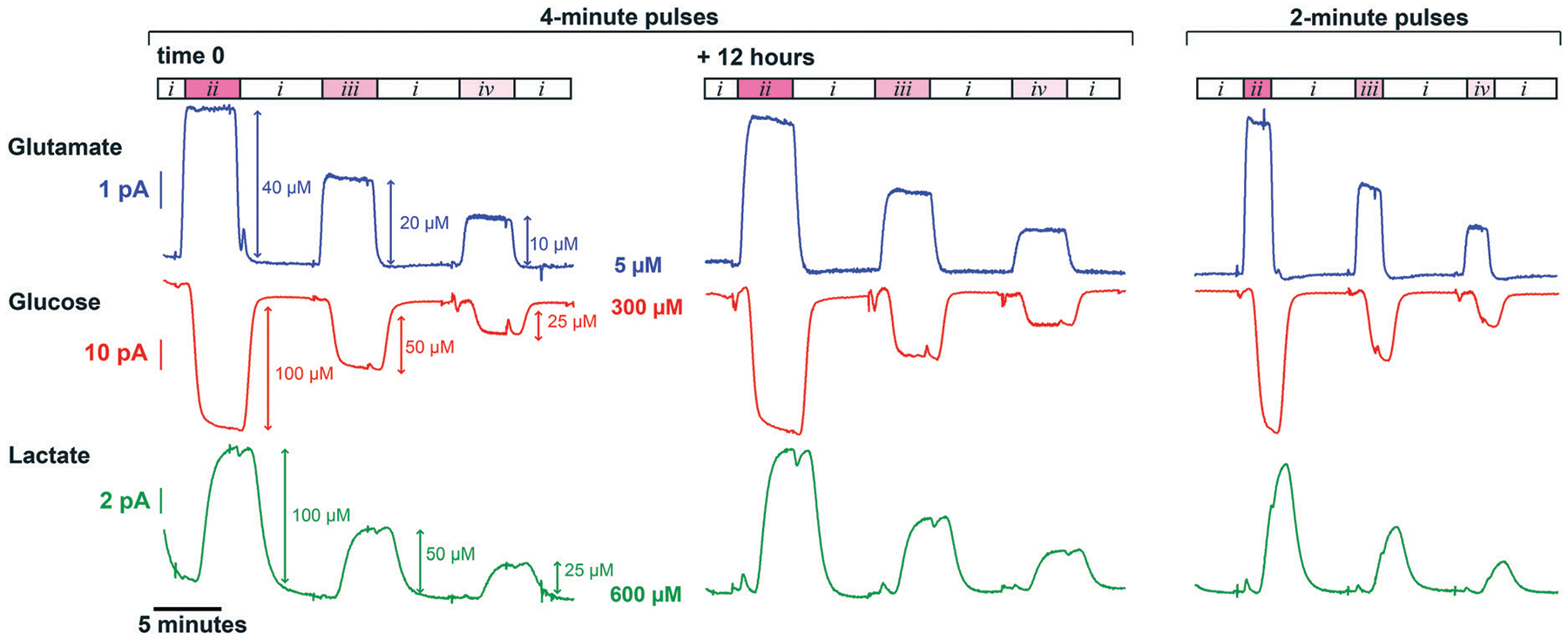
*In vitro* simulation of neurochemical changes mimicking those occurring during SD events. The sequence described below was generated at 2 μl min^−1^ using the microfluidic workstation. Initially, baseline levels of 5 μM glutamate, 300 μM glucose and 600 μM lactate (i) were recorded, after which 4-minute and 2-minute pulses of the three following solutions were generated: 45 μM glutamate, 200 μM glucose and 700 μM lactate (ii), 25 μM glutamate, 250 μM glucose and 650 μM lactate (iii) and 15 μM glutamate, 275 μM glucose and 625 μM lactate (iv). Currents were recorded for a glutamate sensor (position 1), a glucose sensor (position 2) and a polyurethane-coated lactate sensor (position 3) in the microfluidic channel (chip 2). A 51-point Savitzky–Golay filter was applied to all three signals. The sequence was run continuously for 24 hours and the graph shows two 4-minute pulse sequences recorded 12 hours apart, illustrating the analytical robustness of the biosensing platform. In the case of the 2-minute pulse sequence, the plateau is not reached due to dispersion of the analyte down the microfluidic channel. This is particularly noticeable on the lactate trace as the sensor is in position 3 and its response is slower due to the diffusion-limiting polyurethane layer. The artefacts seen are due to the microfluidic pumps refilling and the initial decrease in the baseline of the lactate current is the effect of the charging current.

SD-like physiological events were simulated *in vitro* using the microfluidic workstation and recorded with our high time resolution analyser incorporating sensors for glutamate, glucose and lactate. Glucose and lactate levels were mimicked based on SD events previously recorded in brain dialysate of TBI patients by Rogers *et al*.^24^ This study showed that the simultaneous drop in dialysate glucose and rise in lactate levels triggered by an SD wave occurred on a 5-minute timescale and a 100 μM-amplitude scale on average. Transient extracellular glutamate increases occurring with the onset of an SD have been measured in animal models on a 10 μM scale.^48^ Basal tonic levels of brain extracellular glutamate vary greatly depending on the measurement technique employed but *in vivo* microdialysis and voltammetry studies have recorded concentrations between 1 and 30 μM, with the majority between 1 and 5 μM.^49^ Fig. 4 demonstrates measurements from our novel analyser for simultaneously generated rapid changes in glutamate, glucose and lactate concentrations mimicking an SD physiological response. The graph shows signals that are clearly resolved and stable for over 12 hours for all 3 analytes illustrating the analytical robustness of the analyser. Using simulated dialysate physiological baselines of 5 μM glutamate, 300 μM glucose and 600 μM lactate, the smallest concentration change detectable with the analyser (taken as the concentration corresponding to 3 standard deviations of the baseline current) was 0.6 μM for glutamate, 0.9 μM for glucose and 0.7 μM for lactate. Furthermore, the average *T*_90_ of the 10 μM glutamate rises displayed in Fig. 4 is 23 ± 3 s, which is on the same order of magnitude as behaviourally-induced glutamate changes,^50^ making the system a promising tool to study the neurochemical mechanisms behind decision making.

### Patient monitoring

**Fig. 5:**
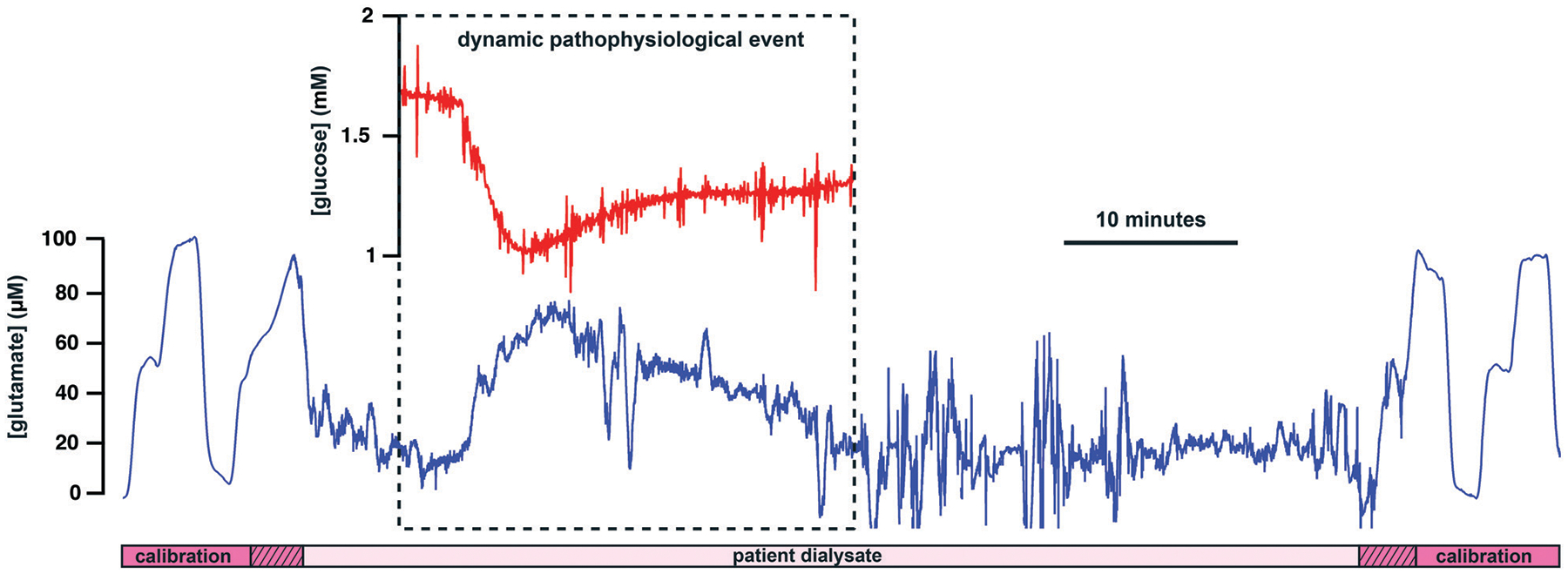
Patient data collected in real time in the ICU using the new lab-on-a-chip device. The blue trace shows dialysate brain glutamate levels bracketed by two automated biosensor calibrations. The bottom bar indicates sections of patient dialysate data (light pink box) and of calibration data (dark pink boxes). The hatched boxes indicate artefacts caused by the microfluidic workstation valves switching from calibration to dialysate and *vice versa*. The red trace in the inset shows dialysate brain glucose levels at the time of the glutamate change, highlighting a dynamic pathophysiological event. The increased level of noise after the dynamic event is electrical noise caused by the patient having their head moved by nursing staff. Dialysate and calibration flow rates are both 2 μl min^−1^.

As a proof-of-concept experiment, our new lab-on-a-chip device was used to monitor a patient with an intracerebral haemorrhage (ICH). Brain chemical monitoring was carried out for 4 days continuously using the device described here. Local glutamate, glucose and lactate levels were monitored successfully. Calibration of the sensors in the microfluidic channel was performed automatically every two hours, a period chosen as a balance between calibration integrity and loss of clinical data. This showed that all three sensors were working during their whole period of use, demonstrating the mechanical and analytical robustness of the device. Fig. 5 shows glutamate data recorded on day 4 of the monitoring period. The sensor calibration cycles on either side of the patient dialysate data validate the full working state of the sensing platform. These calibrations are one-hour apart (instead of the usual 2-hour interval) because a dialysate event (Fig. 5) was observed and therefore a calibration was manually carried out to improve data accuracy. Throughout the monitoring period, glutamate dialysate levels appeared relatively stable and basal levels were found to be 22 ± 6 μM (mean ± standard deviation, *n* = 7 10-minute measurements spread across the recording period), a high level compared to previous reports.^49^ During the first 3 days of monitoring, glucose levels were 0.4 ± 0.1 mM (mean ± standard deviation, *n* = 7), which is on the lower end of the range previously measured in injured human brains.^24^ On day 4, glucose levels were elevated to 1.5 ± 0.5 mM (mean ± standard deviation, *n* = 3). Throughout the whole monitoring period, lactate levels were stable at 1.6 ± 0.4 mM (mean ± standard deviation, *n* = 7), which is high compared to what was previously recorded in patients.^24^ All three metabolite levels recorded here suggest the microdialysis probe was positioned in injured brain tissue. The patient was also monitored for electrophysiology and no SD event was detected in the whole dataset. However, a spontaneous rise in glutamate concentration was detected on day 4 of the monitoring period and this data is shown in Fig. 5. This is the first time a dynamic change in human brain glutamate levels is detected online. This event occurred synchronously with a drop in glucose levels, as shown in the inset of Fig. 5. Taking account of the 29-second transit delay between the two biosensors in the microfluidic channel, the two changes take place at the same time. In addition, they happen on a physiological timescale and the two analyte levels move in opposite direction, enhancing the reliability of the event.^6^ These chemical changes, although looking characteristic of a secondary insult to the brain, did not coincide with an ECoG event (such as an SD), or with a transient change in arterial blood pressure. Although the origin of this event is unknown, it may reflect a different sort of secondary insult to the injured human brain. Indeed, a drop in extracellular glucose levels typically reflects an unmet energy demand while an increase in glutamate levels could be associated with pathophysiological membrane depolarisation.^51^

### Microdialysis experiments

Unlike our previous PDMS chip, the new 3D printed analyser is not affected by movement of the device as the sensors are securely enclosed and locked in the channel. Hence, coupled with wireless electronics, the mechanical robustness and small size of this platform would allow it to be positioned closer to the patient, reducing the length of dialysate tubing required to connect the probe outlet to the analyser, minimising dispersion of concentration changes and improving the temporal resolution within the clinic further still. It would also reduce the delay with which physiological events are detected, allowing the medical team to make critical decisions earlier to potentially minimise the spread of the injury and to prevent the development of secondary brain injury.

To evaluate these potential improvements on delay and temporal resolution, a clinical microdialysis probe was placed in a stirred beaker in which the lactate concentration was increased from 0 to 2 mM. The outlet of the microdialysis probe was connected to the analyser *via* tubing extensions of different lengths. Table 2 shows the delay for the system to detect the start of the event and the *T*_90_ response time for the lactate change in two configurations with different connection tubing lengths. The first configuration “by patient bedside” reproduces the current hospital setup where the analyser is placed on a trolley, connected to the probe outlet *via* a 1 m length of extension tubing (120 μm i.d.). In the “by patient head” configuration, the probe outlet tubing was cut so that the analyser was 5 cm away from the point where the probe would exit the brain, simulating a situation where the analyser would be placed on or around the patient’s head. As shown in Table 2, such a setup would allow detection of physiological events as early as 1.5 minutes after an event occurs and would reduce the *T*_90_ system response time by 48%. Importantly, for clinical use the standard deviations are much smaller, making the system more reproducible.

**Fig. 6:**
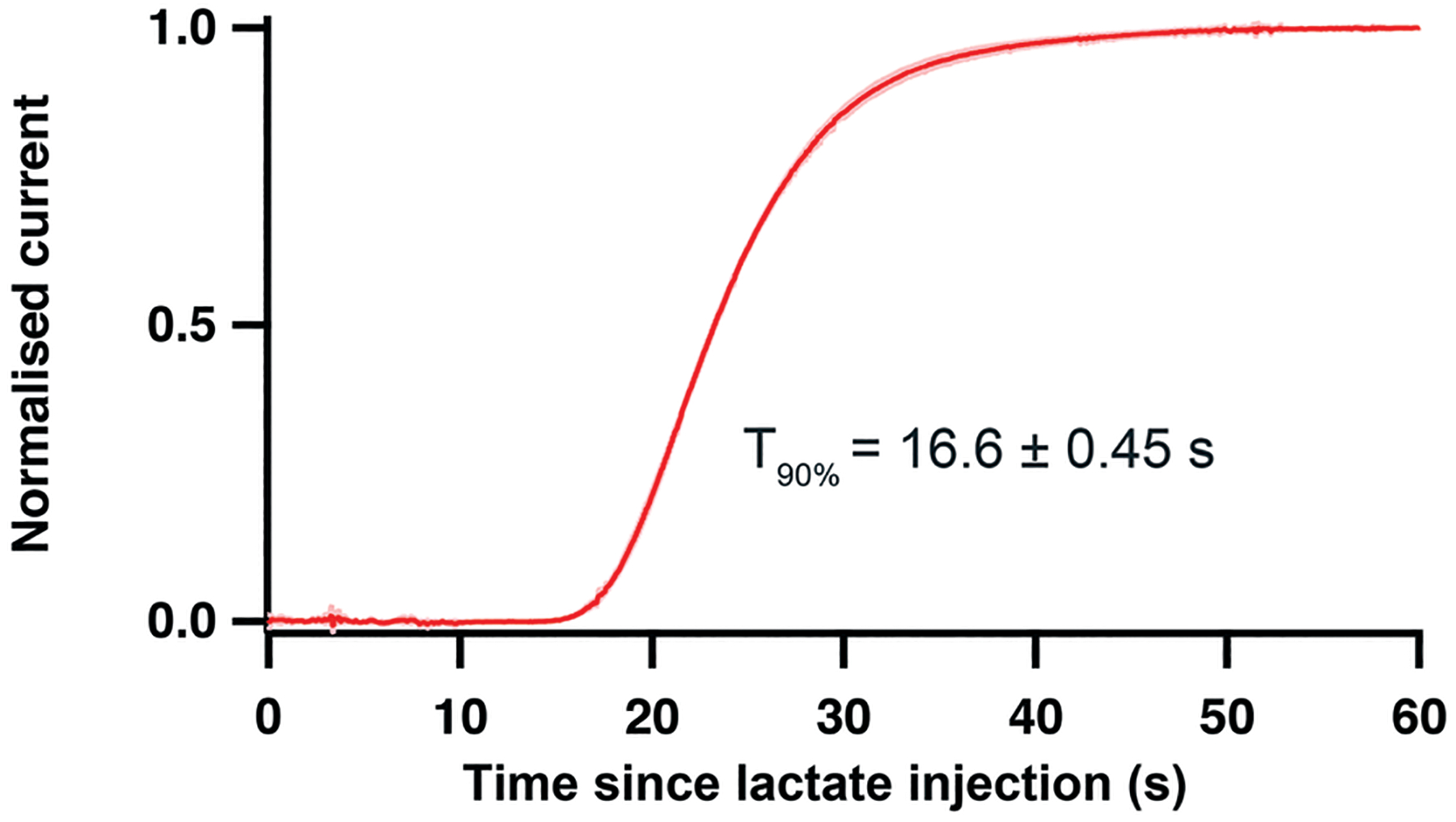
Normalised current response of a lactate sensor placed in the microfluidic channel of chip 2 (position 1) connected to the outlet of an in-house built microdialysis probe (75 μm i.d. outlet tubing) placed in a stirred beaker where a 0 to 2 mM lactate change was induced. Mean response of 3 repeats, standard deviation curves shown in light pink.

The same experiment was also carried out with an in-house built microdialysis probe in the “by patient head” configuration to evaluate the performance of our optimised flow-cell analyser with a sampling system that has a lower outlet-tubing diameter and hence a higher temporal resolution. The current response generated from the lactate concentration increase is shown in Fig. 6. This high-speed system showed a delay as short as 15 s to the onset of sensor response and an improved *T*_90_ response time of 16.6 ± 0.45 s. This test proves that the performance of the system is now being limited by the clinical microdialysis probe. When our analyser is coupled with a low-volume in-house built microdialysis probe instead, it is significantly faster. Indeed, this extremely high time resolution integrated device would enable the detection of physiological events occurring in the tissue surrounding the probe within half a minute.

## Conclusions

We have presented the development and optimisation of a new, mechanically and analytically robust, 3D printed microfluidic analyser for monitoring of dynamic concentration changes in a flow stream with high temporal resolution. Using this new device it is possible to resolve transient metabolite concentration changes as short as 8 s. This high time resolution is perfectly suited for monitoring the dynamic changes in glucose and lactate seen in the injured brain;^38^ it even approaches the fast timescale of SD-induced transient glutamate changes recorded using implanted biosensors.^48^ Simultaneous rapid changes in physiological levels of glutamate, glucose and lactate mimicking dialysate levels seen during SD events were successfully recorded. As a proof-of-concept experiment, the device was used in the ICU to monitor a patient with severe acute brain injury for 4 days continuously, demonstrating the suitability of the device for long-term monitoring. For the first time, a dynamic glutamate change was detected in a living human brain in real time. By careful design, it was possible to incorporate simple screw-type connections for inlet/outlet tubings and to secure sensors reproducibly in the microfluidic channel making the device easy to operate by a non-expert, ideal for clinical monitoring. The mechanical robustness of this new device also allows analysis to occur closer to the patient, removing the need for a bedside trolley and minimising dispersion down the connection tubing. Results presented here show that this would drastically reduce the delay of the system and improve the temporal resolution. The portability of the device is a huge advantage for its use in the ICU where the current wired monitoring systems prevents patient mobility and is cumbersome around routine clinical care. This device shows great promise for clinical monitoring and has potential for use in numerous clinical applications.

## Supplementary Material

SI

## Figures and Tables

**Table 1:** Chip 3D printing parameters and dimensions of printed parts. Measured height and width are the mean ± standard deviation of 10 sections along the channel of chip 1 and 2 measured from CT scan images using ImageJ photoanalysis software. Measured dimensions of chip 3 are from a previous study^23^

Chip	Printer	Material	Printing orientation	Design channel sizes *H* × *W* (μm)	Measured channel height (μm)	Measured channel width (μm)
1	Objet 30	Veroclear	Channel horizontal	170 *×* 100	140 ± 12	130 ± 16
2	Ultra 3SP	ABS 3SP white	Channel vertical	350 *×* 350	209 ± 26	230 ± 26
3	Ultra 3SP	ABS 3SP white	Channel horizontal	520 *×* 520	375 ± 2	508 ± 21

**Table 2:** Improvement in temporal resolution due to the ability of the new chip to be positioned closer to the patient. A clinical microdialysis probe was placed in a stirred beaker and connected to the microfluidic channel (chip 2) holding a lactate sensor (position 1) *via* either a 120 cm length of tubing in the “by patient bedside” configuration (20 cm of probe outlet tubing + 100 cm of FEP extension tubing (i.d. 120 μm)) or *via* a 5 cm length of tubing in the “by patient head” configuration (3 cm of probe outlet tubing + 2 cm of FEP extension tubing). A 0 to 2 mM increase in lactate was created; the delay to the start of the rise in current and *T*_90_ were measured (mean ± standard deviation, *n* = 3)

	Delay (minutes)	*T*_90_ (s)
By patient bedside	7.99 ± 0.24	52.0 ± 9.2
By patient head	1.55 ± 0.07	27.2 ± 0.2

## Footnotes

† Electronic supplementary information (ESI) available. See DOI: 10.1039/c9lc00044e
