# Supplementary material for: 3D printed microfluidic device for online detection of neurochemical changes with high temporal resolution in human brain microdialysate
Source: Lab Chip. Author manuscript; Available in PMC 2022 Jun 21. (PMC9209945; doi:10.1039/c9lc00044e)
Supplement: SI [file NIHMS1816373-supplement-SI.pdf]

Electronic Supplementary Material (ESI) for Lab on a Chip.
This journal is © The Royal Society of Chemistry 2019

Supplementary information

Biosensor fabrication

The following enzyme solutions were used to make biosensors: for glucose biosensors 60 mg/ml glucose oxidase, 30 mg/ml bovine serum albumin (BSA), 20 mg/ml poly(ethylene glycol) diglycidyl ether (PEGDE), 1% glycerol; for lactate biosensors 60 mg/ml lactate oxidase, 30 mg/ml BSA, 20 mg/ml PEDGE, 2% glycerol and for glutamate biosensors 50 mg/ml glutamate oxidase, 81 mg/ml BSA, 78 mg/ml PEDGE, 1% glycerol. All three solutions were made in 10 mM PBS. The electrodes were dipped into the enzyme solutions before being placed in the oven for 2 h at 55°C for glucose and lactate biosensors and for 1.5 h at 45°C for glutamate biosensors.

Microdialysis probe fabrication

In-house concentric microdialysis probes were constructed using Spectra/Por microdialysis hollow fibers (200 µm i.d., 280 µm o.d., 13 kDa MWCO, regenerated cellulose, Spectrum Laboratories, Inc), Smiths Medical Portex inlet tubing (0.38 mm i.d., 1.09 mm o.d) and fused silica outlet tubing (75 µm i.d., 150 µm o.d.) were sealed onto a fused silica shaft (250 µm i.d., 350 µm o.d., Polymicro Technologies, CM scientific) using 2-Ton epoxy (ITW Devcon).

PDMS chip used in current clinical monitoring setup

Figure S1 shows a picture of the microfluidic biosensor setup currently used for clinical monitoring. Unlike the 3D printed chip, the biosensor is inserted directly in the microfluidic channel without the use of a holder. Although a low internal dead volume, the biosensor / PDMS microfluidic chip combination is much less stable than with the 3D printed device. This setup is further described in: M. L. Rogers, C. L. Leong, S. A. Gowers, I. C. Samper, S. L. Jewell, A. Khan, L. McCarthy, C. Pahl, C. M. Tolias, D. C. Walsh, A. J. Strong and M. G. Boutelle, *J. Cereb. Blood Flow Metab.*, 2017, **37**, 1883–1895.

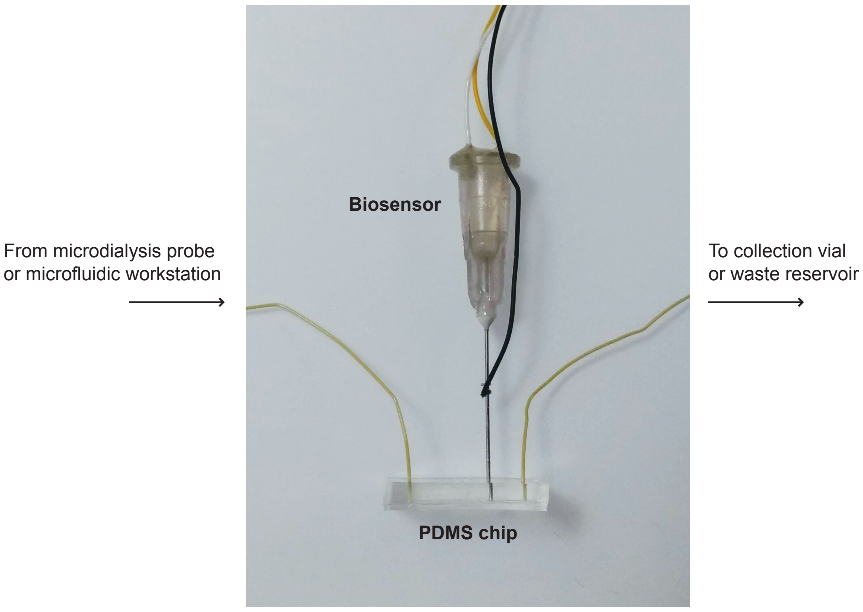

Figure S1. Picture of the PDMS microfluidic chip holding a biosensor, reproducing the measurement setup currently used for clinical monitoring of brain injured patients at King's College Hospital, London.

CT scan images of microfluidic channel

Figure S2a and b shows CT scan images of the microfluidic channels of chip 2 and 3, printed with the Ultra 3SP and with the Objet 30 printers, respectively. The comparison between the two images shows that the channel definition of chip 2 was finer than that of chip 3.

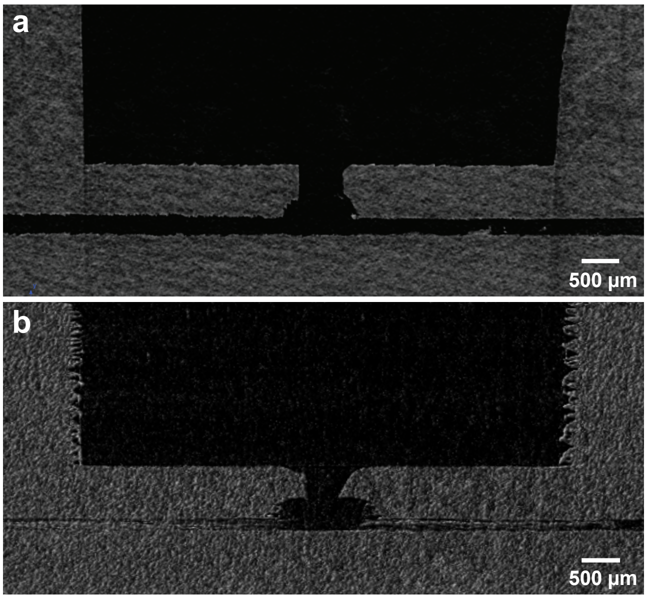

Figure S2. CT scan images of cross sections of channel 2 printed with the Ultra 3SP (**a**) and channel 3 printed with the Objet30 (**b**). **a** shows a very neat straight channel while b shows a distorted channel due to supporting material not cleared evenly (even though this chip was functional).

Figure S3 shows a cross section of chip 2, printed with the Ultra 3SP. The image shows that the microfluidic channel has rounded corners.

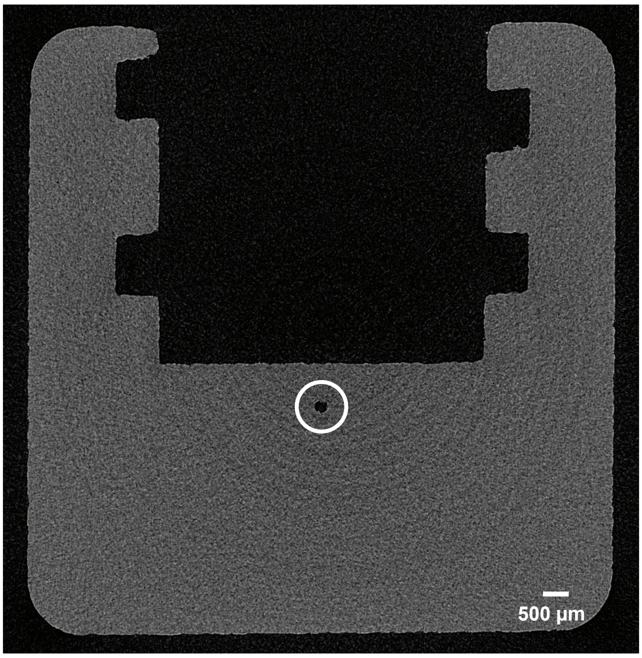

Figure S3. CT scan image of a cross section of chip 2. The white circle shows the quasi-circular microfluidic channel.
